# Postprandial Changes in High Density Lipoproteins in Rats Subjected to Gavage Administration of Virgin Olive Oil

**DOI:** 10.1371/journal.pone.0055231

**Published:** 2013-01-29

**Authors:** Roberto Martínez-Beamonte, María A. Navarro, Sergio Acin, Natalia Guillén, Cristina Barranquero, Carmen Arnal, Joaquín Surra, Jesus Osada

**Affiliations:** 1 Departamento de Bioquímica y Biología Molecular y Celular, Facultad de Veterinaria, Instituto de Investigación Sanitaria de Aragón–Universidad de Zaragoza, Zaragoza, Spain; 2 Departamento de Patología Animal, Facultad de Veterinaria, Universidad de Zaragoza, Zaragoza, Spain; 3 Departamento de Producción Animal, Escuela Politécnica Superior de Huesca, Huesca, Spain; 4 CIBER de Fisiopatología de la Obesidad y Nutrición, Instituto de Salud Carlos III, Madrid, Spain; Paris Institute of Technology for Life, Food and Environmental Sciences, France

## Abstract

**Background and Aims:**

The present study was designed to verify the influence of acute fat loading on high density lipoprotein (HDL) composition, and the involvement of liver and different segments of small intestine in the changes observed.

**Methods and Results:**

To address these issues, rats were administered a bolus of 5-ml of extra-virgin olive oil and sacrificed 4 and 8 hours after feeding. In these animals, lipoproteins were analyzed and gene expressions of apolipoprotein and HDL enzymes were assessed in duodenum, jejunum, ileum and liver. Using this experimental design, total plasma and HDL phospholipids increased at the 8-hour-time-point due to increased sphingomyelin content. An increase in apolipoprotein A4 was also observed mainly in lipid-poor HDL. Increased expression of intestinal *Apoa1*, *Apoa4* and *Sgms1* mRNA was accompanied by hepatic decreases in the first two genes in liver. Hepatic expression of *Abcg1*, *Apoa1bp*, *Apoa2*, *Apoe*, *Ptlp*, *Pon1* and *Scarb1* decreased significantly following fat gavage, while no changes were observed for *Abca1*, *Lcat* or *Pla2g7*. Significant associations were also noted for hepatic expression of apolipoproteins and *Pon1*. Manipulation of postprandial triglycerides using an inhibitor of microsomal transfer protein -CP-346086- or of lipoprotein lipase –tyloxapol- did not influence hepatic expression of *Apoa1* or *Apoa4* mRNA.

**Conclusion:**

All these data indicate that dietary fat modifies the phospholipid composition of rat HDL, suggesting a mechanism of down-regulation of hepatic HDL when intestine is the main source of those particles and a coordinated regulation of hepatic components of these lipoproteins at the mRNA level, independently of plasma postprandial triglycerides.

## Introduction

Several studies have found significant associations between impaired elimination of postprandial lipoproteins and cardiovascular diseases [1], [2]. Triglyceride rich lipoproteins (TRL) observed in the postprandial state are of intestinal or hepatic origin and are referred to, depending on the lipid source, as exogenous or endogenous, respectively [3]. When released from intestine, the lipid core is enveloped by apolipoprotein (APO) B-48 and packaged into chylomicrons (CM). When the source is the liver, lipids engorge a particle containing APOB-100 known as very low density lipoprotein (VLDL). Such clear distribution of apolipoprotein composition reflecting exogenous and endogenous sources of TRL in humans, cannot be extended to rodents due to the fact that their livers produce both apolipoprotein B isoforms [3], [4]. In the periphery, lipoprotein lipase from adipose and muscle tissues releases fatty acids and converts TRL into remnant particles that should be cleared by the liver [5]. These tissues and organs are gatekeepers [6] that regulate postprandial lipemia and potential targets for regulation in response to a great variety of stimuli such as hormones, feeding schedules, composition of foods, etc [6], [7], [8], [9], [10].

High density lipoproteins (HDL) are produced in liver and intestine and to a certain extent, these lipoproteins may be metabolic products of CM and VLDL as observed in knockout mice for intestinal apolipoprotein B and for lipoprotein lipase genes. The latter mice had no HDL when lipoprotein lipase was completely missing, and the particles were produced when the activity was restored after expression the enzyme was achieved in muscle [11]. A genetic model for absent chylomicron formation in mice in which APOB was not expressed in intestine also resulted in low HDL cholesterol levels [12]. These close metabolic relationships among CM, VLDL and HDL demonstrate that HDL may be subject to postprandial regulation, a possibility that needs to be tested in different experimental settings. In addition, several HDL apolipoproteins (eg APOA1, APOA4) are expressed in organs such as liver and intestine [13], and the cross-talk between them to sustain a coordinated response also should be explored in depth considering the complexity of HDL lipoparticles [14].

In rats, due to the absence of cholesteryl ester transfer protein (CETP), most of the plasma cholesterol is transported in HDL [15], -an activity found to parallel postprandial triglyceride response- [16]. Therefore, this model represents a good approach to the study of changes in the postprandial state without the interference of the aforementioned protein and an anticipatory scenario of metabolic changes in humans treated with CETP inhibitors [17] or those lacking this enzyme [18]. Indeed, these subjects showed increased APOA1 and HDL cholesterol levels, mainly corresponding to esterified cholesterol [19], in agreement with the kind of particles also observed in rodents [18]. In addition, rat lipoprotein metabolism has been found to be sensitive to chronic dietary fat amount and composition [20], [21]. In previous experiments in rats, we have shown that a bolus of 16 ml olive oil/kg was sufficient to induce their plasma postprandial response and hepatic lipids and modify the hepatic transcriptome [22], which indicated that this could be a promising approach for testing the hypothesis that an olive oil bolus can influence postprandial HDL composition and regulation. To do so, rats were subjected to gavage administration of virgin olive oil and sacrificed at different time points. In these animals detailed analyses of HDL lipoproteins and apolipoprotein gene expression changes in duodenum, jejunum, ileum and liver were carried out in order to characterize tissue-specific mechanisms involved in the postprandial regulation exerted by an acute intake of fat.

## Results

### Hepatic lipids

**Figure 1:**
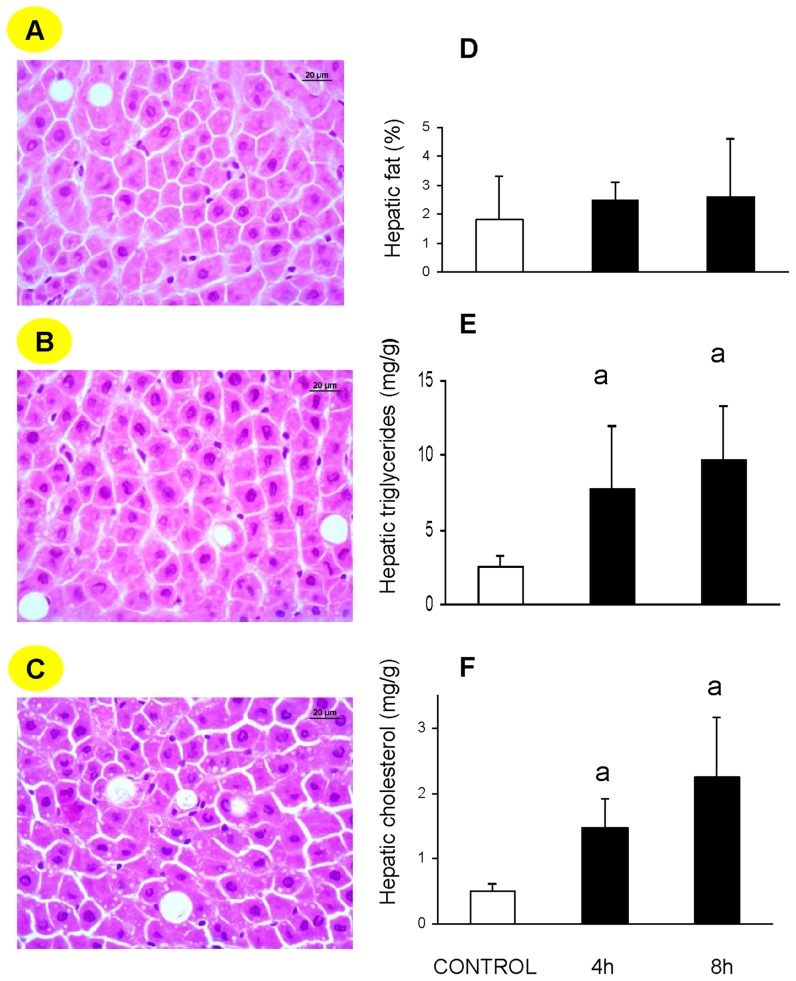
Changes in hepatic steatosis in rats after gavage administration of fat in the form of virgin olive oil. Representative micrographs (×400 magnification) of livers from fasted animals (A) and 4 hours (B) and 8 hours (C) after receiving 5 ml of olive oil as a bolus. Liver sections (4 µm) were stained with hematoxylin and eosin and evaluated blindly. Morphometric changes in hepatic fat content (D) and analysis of triglyceride (E) and cholesterol (F) contents in rats where data are expressed as means ± SD for each group. Statistical analysis to evaluate dietary response was done using one-way ANOVA and the Mann-Whitney U test as post hoc test. ^a^, P<0.05 vs control and ^b^ P<0.05 vs 4 h.

Representative liver micrographs from different experimental groups are shown in Fig. 1A–C. Quantitative morphological evaluation of the percentage of lipid droplets in all animals is depicted in Fig. 1D, which reveals no significant change. In contrast, hepatic triglyceride (TG) content, shown in panel E, increased significantly four hours after gavage with olive oil, and its levels remained significantly elevated at the eight-hour time point. No significant change was observed between the two time periods. Hepatic cholesterol (Fig. 1F) was significantly increased at both time points. Therefore, this experimental design constitutes an interesting approach for the study of the presence of transitory postprandial increase in fat content in liver, probably inside the reticulum, without accumulation of lipid droplets.

### Postprandial plasma lipids and lipoproteins

**Figure 2:**
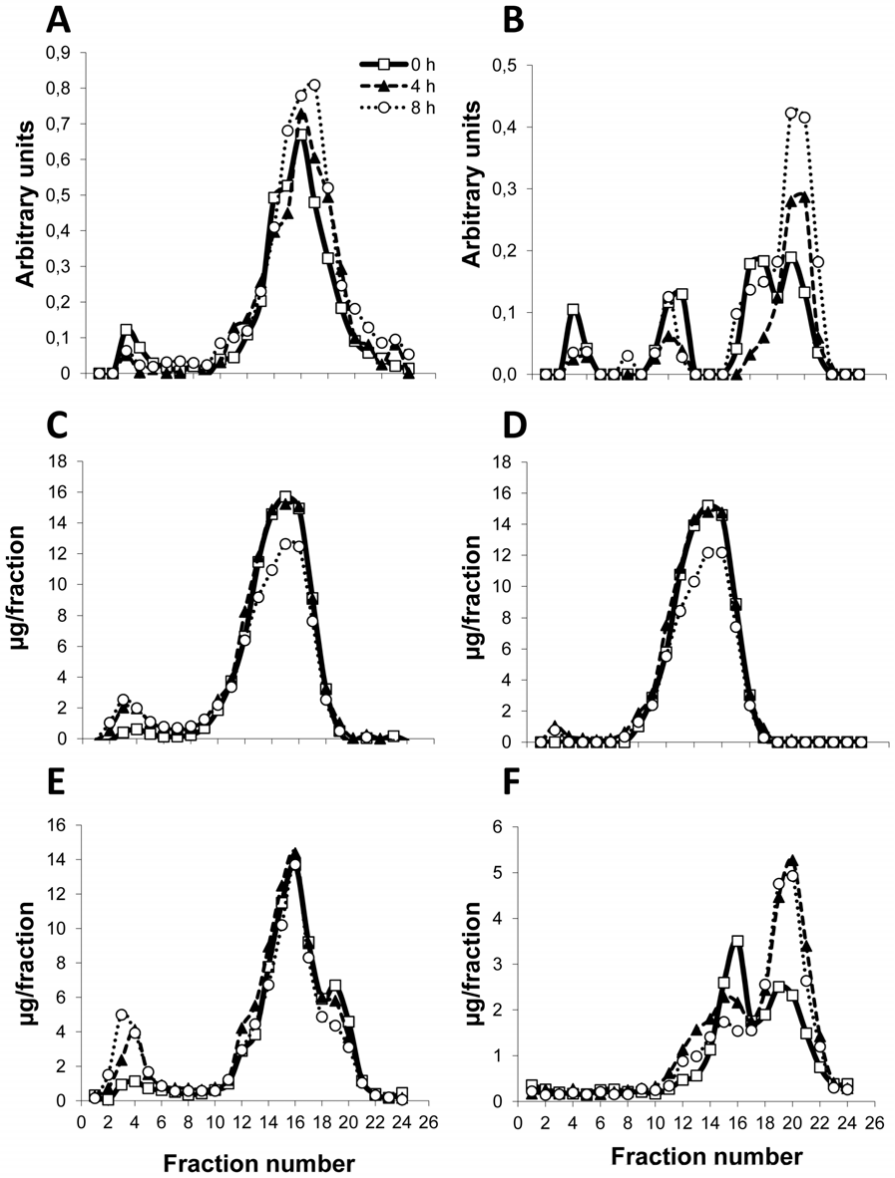
Changes in postprandial lipoproteins in rats after gavage administration of fat in the form of virgin olive oil. After a 16-h fast, male rats were subjected to oral gavage with virgin olive oil (16 ml kg^−1^). Plasma lipoproteins were separated by FPLC and collected fractions analyzed for APOA1 (A), APOA4 (B), total cholesterol (C), esterified cholesterol (D), phosphatidylcholine (E) and sphingomyelin (F). A representative profile of one animal is shown. Fraction numbers 1–6 corresponded to VLDL/chylomicron remnants, 7–13 to low density lipoproteins, 14–18 to cholesterol-rich HDL and 19–24 to cholesterol-poor HDL.

Table 1 shows total plasma lipid levels of rats four and eight hours after gavage with 5 ml of virgin olive oil. Plasma triglycerides were significantly increased under both experimental conditions while plasma cholesterol was not modified at either time point. Phospholipids did not experience any change at the first time point, but increased significantly eight hours after the gavage. As phospholipids are important components of HDL lipoproteins, the increase observed at the 8-hour time point suggests that these particles may have undergone important changes. To address this issue, HDL from rats receiving the olive oil were isolated and characterized. To test the quality of HDL, equal amounts of protein were loaded onto a denaturing gel and electrophoresed. Prepared HDL were devoid of albumin contamination (data not shown) and did not need to be refloated. Compared to the findings in fasted rats (Table 2), there were no significant changes in cholesterol or in triglycerides in these particles. However, HDL phospholipid content was significantly increased, in agreement with the total values in plasma (Table 2). When the lipoproteins were separated by FPLC and analyzed for several components (Figure 2), no changes were observed for APOA1 (Fig. 2A), total cholesterol (Fig. 2C), esterified cholesterol (Fig. 2D) or phosphatidylcholine (Fig. 2E). However, there were increases in APOA4 (Fig. 2B) and sphingomyelin (Fig. 2F), both in lipid-poor HDL particles.

**Table 1:** Time follow-up of rat plasma lipids following the intake of 5 ml virgin olive oil as a fat gavage.

Group\ compounds	Control (n = 5)	4 h (n = 5)	8 h (n = 5)
Cholesterol (mg/dl)	70±4	66±4	70±12
Phospholipids (mg/dl)	85±15	62±15	155±23^a,b^
Triglycerides (mg/dl)	8±2	23±1^a^	40±10^a^

Values are expressed as means ± standard deviations. Statistical analysis was done using non-parametric one-way ANOVA according to the Kruskal-Wallis test and unpaired Mann-Whitney U test as post-hoc test. Superscripts (^a^ vs Control, ^b^ vs 4 h) indicate statistically significant differences (P<0.05).

**Table 2:** Lipid composition of rat HDL following the consumption of 5-ml bolus of virgin olive oil.

Group\ compounds	Control (n = 5)	8 h (n = 5)
Cholesterol (mg/protein g)	128±19	151±12
Phospholipids (mg/protein g)	101±15	178±23^a^
Triglycerides (mg/protein g)	0.9±0.1	0.9±0.1

Values are expressed as means ± standard deviations of 2 pools per group. Statistical analysis was done using unpaired Mann-Whitney U test as post-hoc test. Superscripts (^a^ vs Control) indicate statistically significant differences (P<0.05).

### RNA analysis

To verify the tissue involvement and the potential coordinate expression of apolipoproteins in several organs; liver, duodenum, jejunum and ileum were analysed for their mRNA expressions. Data in Tables 3 and 4 are expressed as arbitrary units referred to the level of the appropriate reference gene [23]. Using this experimental setting, a differential expression among intestinal regions was observed. The most pronounced increases in genes were observed in duodenum for *Apoa1*, *Apoa4 and Sgms1* and the latter two showing a time-dependent increase. In jejunum, there were significant increases only in *Apoa4*, with no significant changes between the two time points studied. No significant changes were observed in ileum (Table 3). In liver (Table 4), significant decreases were observed in *Apoa1*, *Apoa2* and *Apoa4* expression after gavage at both time points. Likewise, decreases were also found for *Apoe* and *Apoa1bp*, although they were only statistically significant 8 hours after the fat administration. Expressions of enzymes phospholipase A2, group VII (platelet-activating factor acetylhydrolase, *Pla2g7*) and paraoxonase/arylesterase 1 (*Pon1*), involved in HDL anti-oxidant action, were also explored and showed differing trends. While the former did not experience any significant change, the latter was found to be significantly decreased both 4 and 8 hours after the fat bolus. Similar changes were detected for expression of genes associated with lipid droplet dynamics, such as *Syt1* and *Cidec*, with no change in the former and reduced expression of the latter. Expressions of enzymes phospholipid transfer protein (*Pltp*) and lecithin-cholesterol acyltransferase *(Lcat)*, and receptors *Abca1*, *Abcg1* and *Scarb1*, which participate in HDL metabolism, were also studied. While *Lcat* and *Abca1* expression did not experience any significant change, *Abcg1*, *Pltp and Scarb1* mRNA levels were significantly decreased after gavage at both time points.

**Table 3:** Changes in gene expression in small intestine fragments following gavage of 5 ml of virgin olive oil.

	Duodenum			Jejunum			Ileum		
Group\ Genes	Control	4 h	8 h	Control	4 h	8 h	Control	4 h	8 h
*Apoa1*	1.3±0.8	38±49^a^	42±22^a^	1.1±0.5	1.5±0.9	1.3±0.9	1.4±0.5	0.8±0.5	0.7±0.6
*Apoa4*	0.8±0.9	27±38^a^	88±41^a,b^	1.0±0.5	3.6±2.6^a^	2.8±1.8^a^	1.9±1.1	2.2±2.0	2.0±2.0
*Sgms1*	1.1±0.6	20±28^a^	56±15^a, b^	1.5±1.5	4.7±3.7	5.0±3.7^a^	2.1±1.6	2.0±1.5	0.5±0.6^a^

Values expressed as means ± standard deviations. Data represent arbitrary units obtained with the RT-qPCR normalized to *Tbp*, *Ubc* and *Hprt* expressions for duodenum, jejunum and ileum, respectively. Statistical analysis was done using non-parametric one-way ANOVA according to Kruskal-Wallis test and unpaired Mann-Whitney U-test as post-hoc test. Superscripts (^a^ vs Control, ^b^ vs 4 h) indicate statistically significant differences (P<0.05).

**Table 4:** Time follow-up of hepatic gene expression following gavage with 5 ml of virgin olive oil gavage.

Group\ Genes	Control	4 h	8 h
*Abca1*	1.9±2.0	1.8±2.4	1.3±1.1
*Abcg1*	1.1±0.4	0.4±0.3^a^	0.3±0.3^a^
*Apoa1*	1.9±0.7	0.4±0.1^a^	0.7±0.4^a^
*Apoa2*	1.0±0.5	0.5±0.3^a^	0.2±0.2^a^
*Apoa1bp*	1.1±0.5	0.7±0.3	0.4±0.3^a^
*Apoa4*	1.1±0.4	0.4±0.2^a^	0.4±0.3^a^
*Apoe*	1.2±0.9	0.8±0.7	0.3±0.2^a^
*Cidec*	1.1±0.7	0.5±0.2^a^	0.4±0.2^a^
*Lcat*	0.9±0.5	0.6±0.3	0.7±0.3
*Pla2g7*	1.0±0.3	1.3±0.7	1.2±0.4
*Pltp*	1.0±0.3	0.5±0.1^a^	0.3±0.1^a^
*Pon1*	1.2±0.9	0.4±0.2^a^	0.3±0.2^a^
*Scarb1*	1.1±0.5	0.5±0.2^a^	0.3±0.1^a^
*Syt1*	1.0±0.1	1.1±0.1	1.1±0.1

Values are means ± standard deviations. Data represent arbitrary units obtained with the RT-qPCR normalized to *Rn18s*. Statistical analysis was done using non-parametric one-way ANOVA according to Kruskal-Wallis test and unpaired Mann-Whitney U-test as post-hoc test. Superscripts (^a^ vs Control) indicate statistically significant differences (P<0.05).

**Figure 3:**
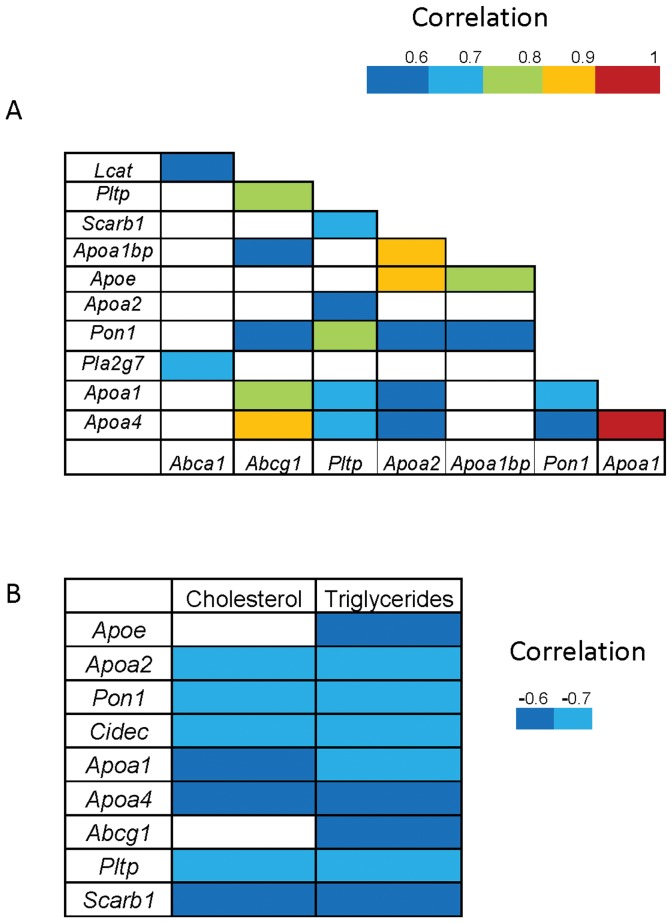
Correlation matrices. Panel A shows the correlations among hepatic mRNA expressions, and panel B represents those found among hepatic mRNA expressions, cholesterol and triglycerides. Correlations were calculated according to the Spearman test.

To verify whether the response of these gene expressions was co-ordinately regulated, an analysis of the association between gene expressions (Figure 3A) was carried out. Indeed, *Apoa1* expression was associated with that of *Apoa4* (ρ = 0.94, P<0.00), *Abcg1* (ρ = 0.77, P<0.001), *Pltp* (ρ = 0.63, P<0.01), *Pon1* (ρ = 0.63, P<0.01) and *Apoa2* (ρ = 0.51, P<0.05). *Apoa2* was associated with *Apoe* (ρ = 0.86, P<0.00), *Apoa1bp* (ρ = 0.85, P<0.00), *Pltp* (ρ = 0.57, P<0.02), *Pon1* (ρ = 0.57, P<0.03) and *Apoa4* (ρ = 0.51, P<0.05). *Apoa4* was associated with *Abcg1* (ρ = 0.88, P<0.000), *Pltp* (ρ = 0.61, P<0.02) and *Pon1* (ρ = 0.59, P<0.02). *Apoe* was associated with *Apoa1bp* (ρ = 0.78, P<0.001). These associations suggest that the mRNA of all these apolipoproteins may share some regulatory mechanisms under the proposed experimental approach. To further characterize the existence of a common compound, associations were carried out with hepatic cholesterol and triglycerides as well. Hepatic gene messengers (*Abcg1*, *Apoa1*, *Apoa2*, *Apoa4*, *Apoe*, *Pltp*, *Pon1 and Scarb1*) corresponding to HDL components were negatively correlated with hepatic triglycerides while only *Apoa1*, *Apoa2*, *Apoa4*, *Pltp*, *Pon1* and *Scarb1* were negatively associated with cholesterol.

### Effects of CP-346086 and tyloxapol on postprandial serum lipid and mRNA levels

**Figure 4:**
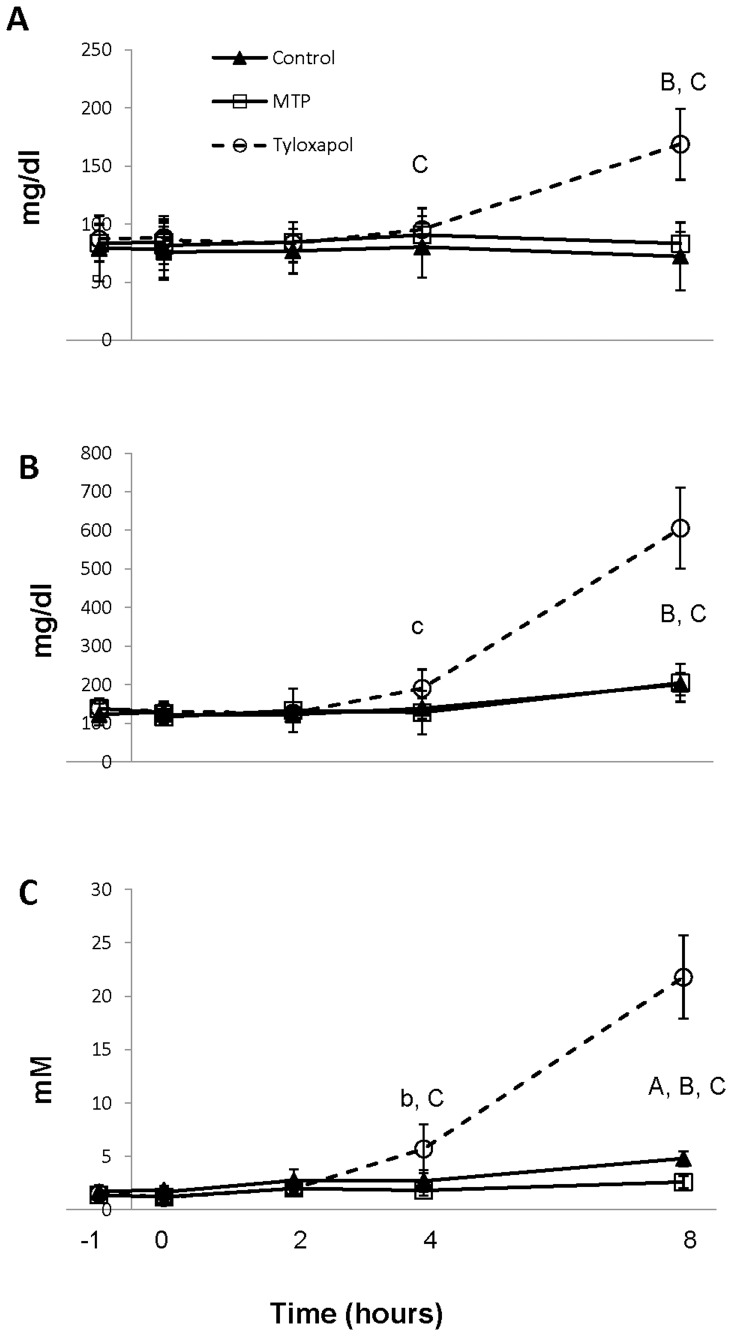
Effect of CP-346086 or tyloxapol administration to rats on postprandial serum lipid levels at different time points. Male rats were fasted overnight and administered vehicle, CP-346086 (500 µg/kg i.p.) or tyloxapol (700 mg/kg i.p.). One hour later, they were subjected to gavage with 5 ml of virgin olive oil, and serum cholesterol (A), phospholipids (B) and triglycerides (C) were determined at the indicated time points. Values are expressed as the mean and SD of 7 animals for each group. Statistical analysis to evaluate dietary response was done using one-way ANOVA and the Mann-Whitney U test as post hoc test. a, p<0.05 and A p<0.01 for MTP vs control; b, p<0.05 and B p<0.01 for tyloxapol vs control; and c p<0.05 and C p<0.01 for MTP vs tyloxapol.

**Figure 5:**
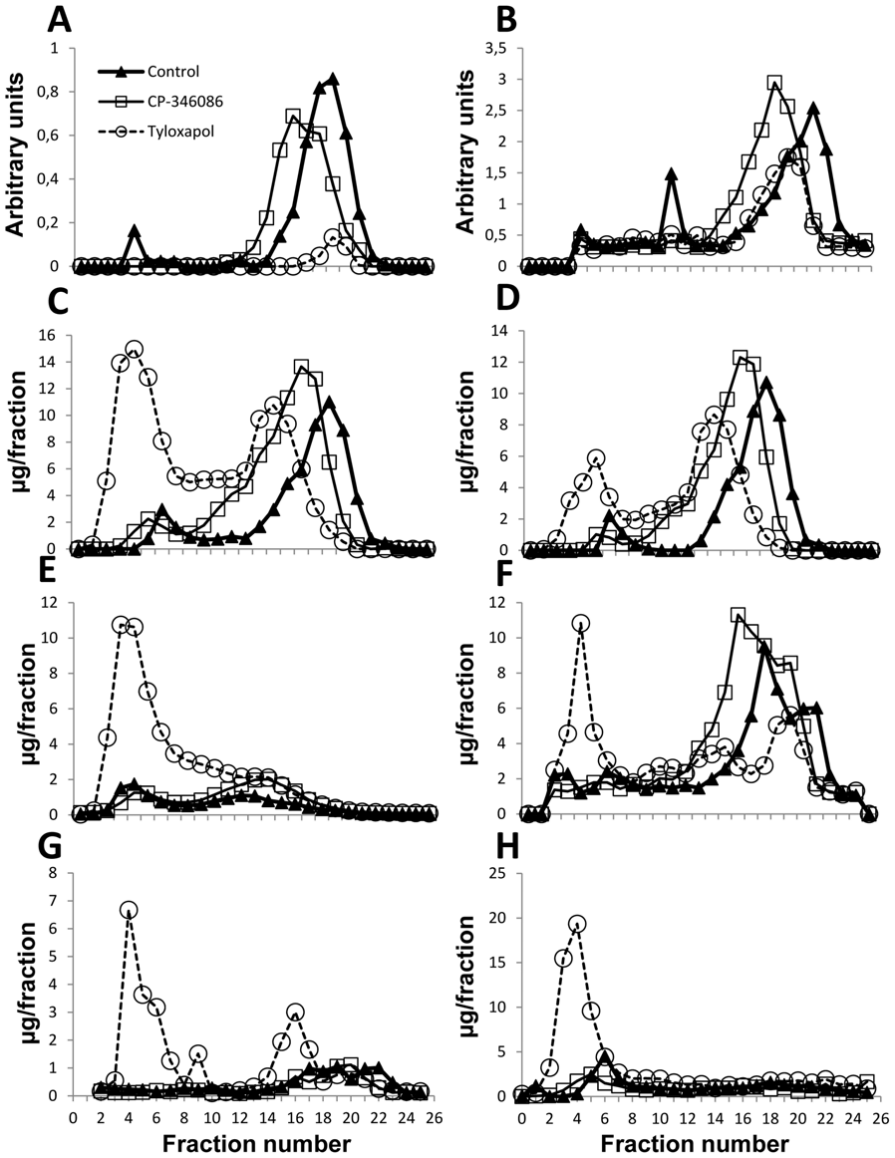
Effect of CP-346086 or tyloxapol administration to rats on postprandial lipoprotein profile at eight hours. Rats were fasted overnight and administered vehicle, CP-346086 or tyloxapol. One hour later, they received a gavage dose of virgin olive oil and were sacrificed 8 hours after feeding. A representative profile of lipoprotein distribution according to APOA1 (A), APOA4 (B), total cholesterol (C), esterified cholesterol (D), free cholesterol (E), phosphatidylcholine (F), sphingomyelin (G) and triglyceride (H) contents.

To explore the transformation of chylomicrons into HDL, two *in vivo* approaches were used: one to inhibit the loading of triglycerides by the action of the microsomal transfer protein (MTP) inhibitor, CP-346086, [24] and the other involving inhibition of plasma lipoprotein lipase by tyloxapol [25]. Using the MTP inhibitor at a dose of 500 µg/kg, a significant reduction in plasma triglyceride concentration occurred 8 hours after gavage with olive oil (Fig. 4 C) with no changes in cholesterol or phospholipids (Fig. 4 A and B). Based on FPLC analysis of lipoprotein at the 8-hour time point, this decrease corresponded to VLDL (Fig. 5 H). Based on the shift to the left observed in the FPLC profile and corresponding to larger particles, CP-346086 treatment induced a larger HDL particle, as revealed by measuring APOA1 (Fig. 5 A), APOA4 (Fig. 5 B), that had a higher amount of total cholesterol (Fig. 5 C), mainly esterified cholesterol (Fig. 5 D), and phosphatidylcholine (Fig. 5 F). No variation in sphingomyelin was observed (Fig. 5G). Taken together, these observations indicate that CP-346086 administration decreases the intestinal triglyceride loading and influences postprandial composition of HDL. These changes in HDL characteristics were not observed in either intestinal (Table 5) or hepatic (data not shown) *Apoa1* or *Apoa4* mRNA expressions.

**Table 5:** Effect of CP-346086 or tyloxapol administration to rats on intestinal gene expression 8 hours after of 5 ml virgin olive gavage.

	Duodenum			Jejunum		
Group\ Genes	Control	CP-346086	Tyloxapol	Control	CP-346086	Tyloxapol
*Apoa1*	1.3±1.6	2.3±2.1	3.1±2.3	1.2±0.7	1.7±1.0	3.8±2.3^a^
*Apoa4*	1.3±1.9	2.3±0.4	3.2±1.9^a^	1.3±1.0	1.5±1.2	2.2±1.4
*Sgms1*	1.6±1.4	2.0±1.3	1.6±1.1	1.0±0.9	1.6±1.4	1.5±1.1

Rats were fasted overnight and administered vehicle (control group), CP-346086 or tyloxapol. One hour later, they received a gavage dose of virgin olive oil and were sacrificed 8 hours after feeding. Values are means ± standard deviations of 7 rats. Data represent arbitrary units obtained with the RT-qPCR normalized to *Tbp* and *Ubc* expressions for duodenum and jejunum, respectively. Statistical analysis was done using non-parametric one-way ANOVA according to Kruskal-Wallis test and unpaired Mann-Whitney U-test as post-hoc test. Different superscripts (^a^ vs Control) are significantly different from each other at P<0.05.

After treatment with tyloxapol, virgin olive oil-fed rats exhibited an accumulation of triglycerides in their plasma that became statistically significant four hours after feeding (Fig. 4 C). Cholesterol and phospholipids showed the same pattern (Fig. 4 A and B). Blocking lipoprotein lipase activity with tyloxapol had a profound impact on APOA1-containing particles, which nearly disappeared (Fig. 5 A), and decreased HDL APOA4 (Fig. 5 B). These changes were independent of intestinal and hepatic mRNA levels (Table 5) since there were significant increases in jejunal *Apoa1* and duodenal *Apoa4* messengers and no changes in hepatic *Apoa1* or *Apoa4* mRNA expression (data not shown). In contrast, accumulation of cholesterol (Fig. 5 C) -mainly as free cholesterol (Fig. 5 E)-, phosphatidylcholine (Fig. 5 F) and sphingomyelin (Fig. 5 G) in VLDL and LDL size fractions explained the increase observed in total plasma levels in rats receiving this agent (Fig. 4). In this experimental approach intestinal *Smgs1* expression was not modified in concordance with plasma sphingomyelin levels (Table 5).

Overall, these experiments indicate that tyloxapol increases plasma triglycerides coming from intestinal loading and that APOA1-containing particles are severely influenced by absence of activity of lipoprotein lipase. This change in HDL characteristics was not associated with hepatic and intestinal *Apoa1* or *Apoa4* mRNA expressions.

## Discussion

The present study was designed to verify the influence of an acute dose of fat, delivered as gastric bolus, on HDL composition at different time points, and the implications of different organs in the possible changes in a model lacking CETP. As expected, plasma triglycerides increased after fat ingestion and, interestingly, HDL showed enrichment of phospholipid content that was reflected in total plasma phospholipids. The increase was mainly due to an increase in sphingomyelin, carried by lipid-poor HDL particles containing APOA4, and associated with increased expression of its Golgi biosynthetic enzyme *Sgms1* in duodenum. Plasma APOA4 levels are reflected in the increased expression of the mRNA of this protein in duodenum and jejunum. Hepatic and intestinal *Apoa1* and *Apoa4* mRNA expressions showed opposing patterns between these organs, and highly associated expressions in liver. Administration of CP-346086 and tyloxapol had little impact on hepatic *Apoa1* and *Apoa4* mRNA expressions despite the profound changes in plasma TG, APOA1 and HDL.

The postprandial state was induced by administration of fat by a gavage dose of virgin olive oil as a single constituent. This approach had been used previously by our group in mice [13] and rats [22], and by others [26], [27], [28], and was well tolerated in both animal models. The present experimental design avoids potential confounding introduced by other components and enables dissection of the effects of the fat, as a single component, on the process [8]. Furthermore, the 16 ml olive oil/kg used, assuming that the rat metabolic rate is tenfold higher than the human, would represent a 1.6 ml/kg and for a 70 kg subject, a supply of 112 g of fat; an amount easily used in postprandial human studies [8]. Our endeavour is particularly relevant to explore phenotype characteristics of models lacking genes where compensatory mechanisms may arise and stressful situations are required [13]. In this regard, the recent incorporation of transgenic and knock-out technology to rat genetics will promote a renewed interest in the use of this animal [29], [30] and our approach may be a useful test for this animal. In contrast, a potential limitation of this approach is the fat bolus accounted for 112% of daily calorie intake, which is not physiological in current nutrition.

The selective enrichment of HDL with phospholipids, together with the modification of fatty acid composition, the latter resembling that of incoming fat (data not shown), and the increased mRNA expressions of *Apoa1* and *Apoa4* in duodenum and jejunum suggest a selective origin from these sources. Further support for this notion is obtained from the decreased mRNA expressions of hepatic *Abcg1*, *Apoa1*, *Apoa2*, *Apoa4*, *Apoe*, *Pltp*, *Pon1* and *Scarb1* particularly as plasma APOA4 was increased mainly in lipid-poor HDL. Moreover, blocking the lipoprotein lipase activity using tyloxapol prior to gavage induced elimination of APOA1-containing particles but not of APOA4. These actions were independent of intestinal mRNA expressions which were increased or not modified (Table 5). The dramatic reduction in APOA1-containing particles would be in agreement with results in lipoprotein lipase knock-out mice [11] and in humans with low activity of this enzyme [31] but the less severe observation for APOA4-containing particles would indicate that not all nascent-particles are equally sensitive to this enzyme action. On the other hand, using CP-346086 as an MTP inhibitor, produced a substantial decrease in plasma TG observed with no change in hepatic and intestinal *Apoa4* gene expressions. Overall, these findings suggest a coordinated regulation between liver and intestine exerted at the mRNA level but independent of plasma triglyceride and intestinal posttranscriptional mechanisms.

Regional differences were observed among intestinal expressions of apolipoprotein gene in response to gavage with virgin olive oil. In this regard, duodenal *Apoa4* expression increased in a time dependent manner (Table 3), while the jejunal increase in terms of this gene message was independent of time. These results are consistent with the data of other authors [26], [32], [33] for the proximal segments of intestine. The basal expression of *Apoa1* and *Apoa4* has been reported to be higher in jejunum than in duodenum and ileum [34]. However, in our experimental approach, duodenum was the site of the greatest increases in the expressions of these genes. Indeed, some authors have claimed a saturation in *Apoa4* jejunal expression at dietary 80 µmol [26], which could explain the less marked response observed in our experimental design using 5500 µmol of triolein. The absence of changes in ileum would be consistent with an ileal blokage executed by nervous signals after fat feeding [35] or compensatory responses of other intestinal segments [36], and would be particularly relevant at triolein intake higher than 1100 µmol [37]. Overall, these data indicate an important role of intestine in providing apolipoproteins for HDL after a fat loading, particularly APOA4. This aspect has been not considered in many studies more focused on APOA4 levels [26], [37], their satiating properties [27], [38], [39] or their antioxidant and antioxidant properties [40], [41]. Another interesting observation from our work is the increase in sphingomyelin in lipid-poor HDL containing APOA4, a finding that reinforces the observation published by Duverger et al. [42] that LpA-IV contained more sphingomyelin, and it supports the notion that this particle is a postprandial phenomenon. When our group cloned the pig SGMS1 gene, we observed a high intestinal expression [43], and we have now found that its intestinal expression increases postprandially. Recently, genetic manipulation of sphingomyelin synthases 1 and 2 has been proved to regulate plasma sphingomyelin levels [44], [45]. Likewise, several environmental conditions have been found to modified plasma sphingomyelin (SM). In this regard, dietary casein significantly raised the amount of sphingomyelin in the VLDL fraction and lowered that of HDL in rats. These findings were explained by enhanced rates of biosynthesis and reduced rates of degradation in the liver. The opposite action was reported when pectin was the source of protein. Dietary cholesterol has also been shown to increase plasma sphingomyelin by down-regulating hepatic activity of acid sphingomyelinase in the liver [46], [47], [48]. Compared to coconut oil, olive oil lowered plasma sphingomyelin levels by enhancing hepatic catabolism [49]. In humans, fish oil supplementation has been shown to increase HDL sphingomyelin [50], [51]. Our data indicate a postprandial rise in sphingomyelin by increasing intestinal *Sgms1* expression. This finding represent a new element of regulation and would be in agreement with the Nilsson and Duan's suggestion that plasma SM increases when plasma lipoprotein pools expand in response to large lipid loads or metabolic abnormalities [52]. Sphingomyelin has proposed to be a physiological inhibitor of cholesterol esterification in the plasma, by virtue of its competition with phosphatidylcholine, the acyl donor for the lysolecithin acyltransferase (LCAT) reaction [53]. It may also inhibit lipoprotein lipase and the interaction of lipoproteins with receptors [52]. These would be in agreement with our data of the tyloxapol experiment when an increase in VLDL/remnant chylomicron sphingomyelin was associated with an increase in free cholesterol (Figure 5). However, the opposite effect of APOA4-containing HDL with higher content of sphingomyelin as a stimulatory of LCAT activity [42]and the cellular efflux [54] has also been reported to. In this regard, our increase in HDL sphingomyelin following the postprandial regimen (Figure 2) was not reflected in changes in esterified cholesterol. Overall, these data are indicating that the source of lipoproteins containing sphingomyelin may be important regarding the global effect of this phospholipid.

The close correlations noted in hepatic gene expressions of *Abcg1*, *Apoa1*, *Apoa2*, *Apoa4*, *Apoe*, *Pltp* and *Pon1* genes add an additional feature of a potential common regulatory mechanism under the forced choice of this experimental design. Interestingly, all these hepatic gene messages were negatively correlated with hepatic triglycerides (Fig. 3B). This fact indicates that these compounds would drive the hepatic transcription of these genes. A particular limitation of this study could be the use of a single dietary component such as olive oil, taking into consideration that other dietary components (vg cysteine) may be involved in HDL and APOA1 regulation [55].

In conclusion, a selective lipid and apolipoprotein composition of HDL is modulated by gavage administration of fat in the form of virgin olive oil. Changes at the mRNA levels in liver, duodenum and jejunum are involved. The opposite nature of the changes in the liver and intestine in terms of *Apoa1* and *Apoa4* points to the latter organ as the main source of lipoproteins responsible for the increase in postprandial plasma APOA4. The decreases observed in hepatic *Abcg1*, *Apoa2*, *Apoe, Pltp*, *Pon1* and *Scarb1* also corroborate this effect. Furthermore, the close correlation among hepatic expressions of all HDL hepatic components suggests an orchestrated regulation of them, not necessarily dependent on plasma triglyceridemia. Further research is necessary to gain more insight into the proposed mechanisms and to achieve a complete picture of them.

## Materials and Methods

### Rats

Male Wistar rats, weighing 250–300 g (purchased from Charles River, Barcelona, Spain), were used for experiments. Rats, housed in sterile filter-top cages (3–4 per cage), were acclimatized in a room maintained at 20°C with a 12-h light-dark cycle for 10 days, allowed *ad libitum* access to water and standard chow diet (Pascual S.A., Barcelona, Spain), and fasted for 18 h before experiments. Animals were handled and killed always observing criteria from the European Union for care and use of laboratory animals in research, and the protocol was approved by the Ethics Committee for Animal Research of the University of Zaragoza.

### Experimental design

#### Postprandial assay

A baseline fasting blood sample was obtained from tail vein. Blood samples were collected in heparin-coated capillary tubes and centrifuged at 2000 g for 5 min. The plasma obtained was maintained at 4°C for immediate triglyceride analysis and used for randomization of rats into three groups of 5 each. The control group did not receive any fat meal. The other two groups were fed 5 ml of extra virgin olive oil (Aceites Toledo, Spain) as a bolus and sacrificed 4 and 8 hours after the feeding, respectively. This amount represents the use of a dose of 16 ml olive oil/kg (112% of daily calories), sufficient to induce a plasma postprandial response in rat and to evaluate absorption without the use of radioactivity [22].The fatty acid composition of the olive oil, shown in Table S1, indicates that oleic acid was the main fatty acid component. Olive oil was administered directly to stomach using a 1.1-mm diameter, 50-mm-long flexible Abbocath connected to a sterile polypropylene syringe and delivered in 4 seconds. At the moment of sacrifice, rats were anesthetized with 1 ml of 8% Avertine (Aldrich Chemical Co., Madrid, Spain) in 0.1 M phosphate, pH 7.2, and blood drawn from hearts. Blood was collected in tubes containing 1 g/l sodium EDTA. Liver and small intestine were removed and quickly frozen in liquid N_2_ until total RNA was extracted.

#### Postprandial assay in presence of lipoprotein lipase and microsomal triglyceride transfer protein inhibitors

An 18-hour-fasting blood sample was obtained from tail vein. Then, three groups of 7 male rats were established and intraperitoneally injected: the first group (control) with 0.5 ml of PBS, the second with an identical volume containing the lipoprotein lipase inhibitor, Tyloxapol (Sigma), to provide a dose of 700 mg/kg per animal [25], and the third group with a solution containing the microsomal triglyceride transfer protein inhibitor, CP-346086 (Sigma) [24], at 500 µg/kg. One hour later, all animals underwent gavage with 5-ml extra virgin olive oil (Aceites Toledo, Spain). Blood samples from tail vein were obtained at 2, 4 and 8-hours post-feeding. At the latter time point, animals were euthanized and liver and small intestine were removed and quickly frozen in liquid N_2_ until total RNA was extracted.

### Lipid and lipoprotein analyses

Total plasma cholesterol, triglyceride (corrected for free glycerol) and phospholipid concentrations were quantified enzymatically in a microtiter assay using commercial kits from Sigma Chemical Co. (Madrid, Spain) and Roche (Barcelona, Spain). Cardiolipid (Sigma) was used as quality control.

Lipoprotein fractions were prepared from 2 pools of fresh plasma samples of (5 animals per group) by ultra centrifugation in a Kontron T-2060 using a Kontron TST 41.14 rotor and based on a combination of the methods proposed by [56] and [57]. First, chylomicrons were removed by ultra-centrifuging 12 ml of plasma at 40,000 rpm for 30 min at 4°C. Then, 2 ml of the CM-depleted plasma were mixed with 0.77 g of NaBr to reach a final density of 1.44 g/ml, and Sudan Black (0.2 mg/ml in dimethyl sulfoxide) was added. Next, a sodium-bromide discontinuous gradient (2.2 ml of d = 1.389, 1 ml of d = 1.210, 3 ml of d = 1.100, 2 ml of d = 1.063, 2 ml of d = 1.019 and 1 ml d = 1.006 g/ml) was carefully layered and used to separate very-low-density lipoproteins, intermediate-density lipoproteins, low-density lipoproteins and high-density lipoproteins by ultracentrifugation (40,000 rpm for 22 h, at 4°C). Ultracentrifuge tubes were sliced, each volume fraction was collected and NaBr eliminated by centrifugation in Centricon tubes number 10, pore size 10K (Amicon Inc. Beverly, MA, USA). The density of the efflux was measured using a refractometer to estimate the buoyant density for each fraction and the values obtained were similar to those previously reported [58]. Concentrations of HDL cholesterol, triglycerides and phospholipids were determined as described above. Protein content was quantified by Bradford's method [59]. Quality of HDL was assessed by electrophoresis of 15 µg of protein fraction on 4–22% polyacrylamide gel, run at 85V for 18 h, and stained with Coomassie brilliant blue-R, or with silver stain [60] when more sensitivity was required. Images were acquired by Gel Doc 1000 and analyzed using Quantity One® software version 4.5.0 (Biorad, Madrid, Spain).

Plasma lipoprotein profile was also determined in 100 µl of plasma samples by fast protein liquid chromatography (FPLC) gel filtration [61] using a Superose 6B column (Amersham Pharmacia, Barcelona, Spain), and the total cholesterol in each fraction was measured using a fluorometric method (Amplex Red, Molecular Probes, USA). Free cholesterol was estimated with the fluorimetric assay omitting cholesterol esterase and esterified cholesterol as the difference between total and free cholesterol forms. Apolipoproteins (APOA1 and APOA4) were quantified by ELISA using specific polyclonal antibodies (Biodesign, Saco, ME, and Santa Cruz Biotechnology, Santa Cruz, CA, USA), as previously described [62]. Phosphatidylcholine and sphingomyelin were determined using the enzymatic procedure of Hojjati et al. [63] coupled to fluorometric detection as described by He et al. [64].

### Histological analysis

A sample of liver from each rat was stored in 4% buffered formaldehyde and embedded in paraffin. Sections (4 µm) were stained with hematoxylin and eosin and observed using a Nikon microscope, and images were captured with Nikon camera. The surface area of lipid droplets was quantified in each liver section with Adobe Photoshop CS2 and expressed as percentage of total liver section [65].

### Hepatic lipid analysis

Tissues (10 mg) were homogenized in 1 ml of PBS. An aliquot was saved to determine protein concentration by the BioRad dye binding assay (BioRad, Madrid, Spain). One volume of homogenate was extracted twice with two volumes of chloroform: methanol (2∶1). The separated organic phases of each animal were combined and evaporated under N_2_ stream. Extracts were dissolved in 100 µL of isopropanol to estimate cholesterol and triglyceride concentrations using commercial kits as mentioned above.

### RNA isolation

RNA was isolated using Tri reagent (Sigma). Contaminant DNA was removed by TURBO DNAse treatment from AMBION (Austin, TX, USA). RNA was quantified by absorbance at A_260/280_ (the A_260/280_ ratio was greater than 1.75). The integrity of samples was verified by the 28S/18S ratio of ribosomal RNAs and the RNA integrity number (Agilent 2100 Bioanalyzer). No significant differences were observed among the groups tested for either index [23].

### Quantification of mRNA

Equal amounts of DNA-free RNA from each sample of each animal were used in reverse transcriptase-quantitative polymerase chain reaction (PCR) analyses. First-strand cDNA synthesis and the PCR reactions were performed using the Power SYBR® Green (Applied Biosystems, Foster City, CA), according to the manufacturer's instructions and as previously described [66]. Primers were designed by Primer Express® (Applied Biosystems) and checked by BLAST analysis (NCBI) to verify specificity and selective amplification of the target gene, as well as to amplify cDNA but not genomic DNA. The characteristics, according to MIQE guidelines [67], are shown in Table S2. Real time PCR reactions were performed in an ABI PRISM 7700 Sequence Detector (Applied Biosystems) following the standard procedure. The specificity of the PCR reaction was confirmed by observing a single dissociation curve. The relative amount of all mRNAs was calculated using the comparative 2^−ΔΔCq^ method. After a careful evaluation of reference genes, *Rn18s*, *Tbp*, *Ubc* and *Hprt* were used to normalize gene expression changes for liver, duodenum, jejunum and ileum, respectively [23].

### Statistical analysis

The results are expressed as means ± SD. Comparisons were made using one-way ANOVA and the Tukey-Kramer multiple comparison test (*post hoc*) when the distribution of the variables was normal. When the variables did not show such a distribution (according to the Shapiro-Wilk test), or failed to show homology of variance, comparisons were calculated by the Mann-Whitney U test. Correlations between variables were sought using the Pearson or Spearman correlation coefficient**s**. SPSS version 15.0 (SPSS Inc, Chicago, IL) and Instat 3.02 software packages for Windows (GraphPad, S. Diego, CA, USA) were used for calculations. Significance was set at P≤0.05.

## Supporting Information

Table S1 Fatty acid composition of virgin olive oil.
(DOCX)

Table S2 Nucleotide sequence of primers used for RT-qPCR according to MIQE guidelines.
(DOCX)

## References

[1] Redgrave TG (2008) Chylomicrons in disease-future challenges Invited keynote address. Atheroscler Suppl 9: 3–6. 18585100 10.1016/j.atherosclerosissup.2008.05.002

[2] Kannel WB, Vasan RS (2009) Triglycerides as vascular risk factors: new epidemiologic insights. Curr Opin Cardiol 24: 345–350. 19424059 10.1097/HCO.0b013e32832c1284 PMC3012388

[3] Iqbal J, Hussain MM (2009) Intestinal lipid absorption. Am J Physiol Endocrinol Metab 296: E1183–E1194. 19158321 10.1152/ajpendo.90899.2008 PMC2692399

[4] Hussain MM, Kedees MH, Singh K, Athar H, Jamali NZ (2001) Signposts in the assembly of chylomicrons. Front Biosci 6: D320–D331. 11229873 10.2741/hussain

[5] Mahley RW, Huang Y, Rall SC Jr (1999) Pathogenesis of type III hyperlipoproteinemia (dysbetalipoproteinemia). Questions, quandaries, and paradoxes. J Lipid Res 40: 1933–1949. 10552997

[6] Cianflone K, Paglialunga S, Roy C (2008) Intestinally derived lipids: metabolic regulation and consequences–an overview. Atheroscler Suppl 9: 63–68. 18693144 10.1016/j.atherosclerosissup.2008.05.014

[7] Perez-Martinez P, Lopez-Miranda J, Perez-Jimenez F, Ordovas JM (2008) Influence of genetic factors in the modulation of postprandial lipemia. Atheroscler Suppl 9: 49–55. 18603482 10.1016/j.atherosclerosissup.2008.05.005

[8] Bergeron N, Havel RJ (1997) Assessment of postprandial lipemia: nutritional influences. Curr Opin Lipidol 8: 43–52. 9127711 10.1097/00041433-199702000-00010

[9] Lairon D (2008) Macronutrient intake and modulation on chylomicron production and clearance. Atheroscler Suppl 9: 45–48. 18595783 10.1016/j.atherosclerosissup.2008.05.006

[10] Xu T, Li X, Ma X, Zhang Z, Zhang T, et al (2009) Effect of diacylglycerol on postprandial serum triacylglycerol concentration: a meta-analysis. Lipids 44: 161–168. 18989717 10.1007/s11745-008-3258-2

[11] Weinstock PH, Bisgaier CL, Aalto-Setala K, Radner H, Ramakrishnan R, et al (1995) Severe hypertriglyceridemia, reduced high density lipoprotein, and neonatal death in lipoprotein lipase knockout mice. Mild hypertriglyceridemia with impaired very low density lipoprotein clearance in heterozygotes. J Clin Invest 96: 2555–2568. 8675619 10.1172/JCI118319 PMC185959

[12] Young SG, Cham CM, Pitas RE, Burri BJ, Connolly A, et al (1995) A genetic model for absent chylomicron formation: Mice producing apolipoprotein B in the liver, but not in the intestine. J Clin Invest 96: 2932–2946. 8675665 10.1172/JCI118365 PMC186005

[13] Maeda N, Li H, Lee D, Oliver P, Quarfordt SH, et al (1994) Targeted disruption of the apolipoprotein C-III gene in mice results in hypertriglyceridemia and protection from postprandial hypertriglyceridemia. J Biol Chem 269: 23610–23616. 8089130

[14] Lou-Bonafonte JM, Fito M, Covas MI, Farras M, Osada J (2012) HDL-Related Mechanisms of Olive Oil Protection in Cardiovascular Disease. Curr Vasc Pharmacol 10: 392–409. 22339299 10.2174/157016112800812827

[15] Overturf ML, Loose-Mitchell DS (1992) In vivo model systems: the choice of the experimental animal model for analysis of lipoproteins and atherosclerosis. Curr Opin Lipidol 3: 179–185.

[16] Tall AR, Sammet D, Granot E (1986) Mechanisms of enhance cholesteryl ester transfer from high density lipoproteins to apolipoprotein B-containing lipoproteins during alimentary lipemia. J Clin Invest 77: 1163–1172. 3958185 10.1172/JCI112417 PMC424452

[17] Hewing B, Fisher EA (2012) Rationale for cholesteryl ester transfer protein inhibition. Curr Opin Lipidol 10.1097/MOL.0b013e328353ef1d PMC3924318 22517614

[18] Barter P, Rye KA (2011) Cholesteryl ester transfer protein inhibition to reduce cardiovascular risk: Where are we now? Trends Pharmacol Sci 32: 694–699. 22088767 10.1016/j.tips.2011.07.004

[19] Clark RW, Sutfin TA, Ruggeri RB, Willauer AT, Sugarman ED, et al (2004) Raising high-density lipoprotein in humans through inhibition of cholesteryl ester transfer protein: an initial multidose study of torcetrapib. Arterioscler Thromb Vasc Biol 24: 490–497. 14739125 10.1161/01.ATV.0000118278.21719.17

[20] Calleja L, Trallero MC, Carrizosa C, Mendez MT, Palacios-Alaiz E, et al (2000) Effects of dietary fat amount and saturation on the regulation of hepatic mRNA and plasma apolipoprotein A-I in rats. Atherosclerosis 152: 69–78. 10996341 10.1016/s0021-9150(99)00451-7

[21] Osada J, Fernandez-Sanchez A, Diaz-Morillo JL, Miró-Obradors MJ, Cebrián JA, et al (1994) Differential effect of dietary fat saturation and cholesterol on hepatic apolipoprotein gene expression in rats. Atherosclerosis 108: 83–90. 7980709 10.1016/0021-9150(94)90039-6

[22] Martinez-Beamonte R, Navarro MA, Guillen N, Acin S, Arnal C, et al (2011) Postprandial transcriptome associated with virgin olive oil intake in rat liver. Front Biosci (Elite Ed) 3: 11–21. 21196280 10.2741/e215

[23] Martinez-Beamonte R, Navarro MA, Larraga A, Strunk M, Barranquero C, et al (2011) Selection of reference genes for gene expression studies in rats. J Biotechnol 151: 325–334. 21219943 10.1016/j.jbiotec.2010.12.017

[24] Chandler CE, Wilder DE, Pettini JL, Savoy YE, Petras SF, et al (2003) CP-346086: an MTP inhibitor that lowers plasma cholesterol and triglycerides in experimental animals and in humans. J Lipid Res 44: 1887–1901. 12837854 10.1194/jlr.M300094-JLR200

[25] Borensztajn J, Rone MS, Kotlar TJ (1976) The inhibition in vivo of lipoprotein lipase (clearing-factor lipase) activity by triton WR-1339. Biochem J 156: 539–543. 949335 10.1042/bj1560539 PMC1163786

[26] Kalogeris TJ, Fukagawa K, Tso P (1994) Synthesis and lymphatic transport of intestinal apolipoprotein A-IV in response to graded doses of triglyceride. J Lipid Res 35: 1141–1151. 7964177

[27] Kalogeris TJ, Rodriguez MD, Tso P (1997) Control of synthesis and secretion of intestinal apolipoprotein A-IV by lipid. J Nutr 127: S537–S543. 10.1093/jn/127.3.537S 9082042

[28] Kusunoki J, Aragane K, Kitamine T, Kozono H, Kano K, et al (2000) Postprandial hyperlipidemia in streptozotocin-induced diabetic rats is due to abnormal increase in intestinal acyl coenzyme A:cholesterol acyltransferase activity. Arterioscler Thromb Vasc Biol 20: 171–178. 10634814 10.1161/01.atv.20.1.171

[29] Cozzi J, Anegon I, Braun V, Gross AC, Merrouche C, et al (2009) Pronuclear DNA injection for the production of transgenic rats. Methods Mol Biol 561: 73–88. 19504065 10.1007/978-1-60327-019-9_5

[30] Geurts AM, Cost GJ, Freyvert Y, Zeitler B, Miller JC, et al (2009) Knockout rats via embryo microinjection of zinc-finger nucleases. Science 325: 433. 19628861 10.1126/science.1172447 PMC2831805

[31] Reymer PW, Gagne E, Groenemeyer BE, Zhang H, Forsyth I, et al (1995) A lipoprotein lipase mutation (Asn291Ser) is associated with reduced HDL cholesterol levels in premature atherosclerosis. Nat Genet 10: 28–34. 7647785 10.1038/ng0595-28

[32] Kalogeris TJ, Tsuchiya T, Fukagawa K, Wolf R, Tso P (1996) Apolipoprotein A-IV synthesis in proximal jejunum is stimulated by ileal lipid infusion. Am J Physiol 270: G277–286. 8779969 10.1152/ajpgi.1996.270.2.G277

[33] Rodriguez MD, Kalogeris TJ, Wang XL, Wolf R, Tso P (1997) Rapid synthesis and secretion of intestinal apolipoprotein A-IV after gastric fat loading in rats. Am J Physiol 272: R1170–1177. 9140017 10.1152/ajpregu.1997.272.4.R1170

[34] Elshourbagy NA, Boguski MS, Liao WS, Jefferson LS, Gordon JI, et al (1985) Expression of rat apolipoprotein A-IV and A-I genes: mRNA induction during development and in response to glucocorticoids and insulin. Proc Natl Acad Sci U S A 82: 8242–8246. 3934672 10.1073/pnas.82.23.8242 PMC391479

[35] Sonoyama K, Tajima K, Fujiwara R, Kasai T (2000) Intravenous infusion of hexamethonium and atropine but not propranolol diminishes apolipoprotein A-IV gene expression in rat ileum. J Nutr 130: 637–641. 10702597 10.1093/jn/130.3.637

[36] Rubin DC (1992) Spatial analysis of transcriptional activation in fetal rat jejunal and ileal gut epithelium. Am J Physiol 263: G853–863. 1476193 10.1152/ajpgi.1992.263.6.G853

[37] Apfelbaum TF, Davidson NO, Glickman RM (1987) Apolipoprotein A-IV synthesis in rat intestine: regulation by dietary triglyceride. Am J Physiol 252: G662–666. 3555115 10.1152/ajpgi.1987.252.5.G662

[38] Tso P, Liu M, Kalogeris TJ, Thomson AB (2001) The role of apolipoprotein A-IV in the regulation of food intake. Annu Rev Nutr 21: 231–254. 11375436 10.1146/annurev.nutr.21.1.231

[39] Glatzle J, Darcel N, Rechs AJ, Kalogeris TJ, Tso P, et al (2004) Apolipoprotein A-IV stimulates duodenal vagal afferent activity to inhibit gastric motility via a CCK1 pathway. Am J Physiol Regul Integr Comp Physiol 287: R354–359. 15117731 10.1152/ajpregu.00705.2003

[40] Spaulding HL, Saijo F, Turnage RH, Alexander JS, Aw TY, et al (2006) Apolipoprotein A-IV attenuates oxidant-induced apoptosis in mitotic competent, undifferentiated cells by modulating intracellular glutathione redox balance. Am J Physiol Cell Physiol 290: C95–C103. 16120654 10.1152/ajpcell.00388.2005

[41] Vowinkel T, Mori M, Krieglstein CF, Russell J, Saijo F, et al (2004) Apolipoprotein A-IV inhibits experimental colitis. J Clin Invest 114: 260–269. 15254593 10.1172/JCI21233 PMC450164

[42] Duverger N, Ghalim N, Theret N, Fruchart JC, Castro G (1994) Lipoproteins containing apolipoprotein A-IV: composition and relation to cholesterol esterification. Biochim Biophys Acta 1211: 23–28. 8123678 10.1016/0005-2760(94)90134-1

[43] Guillen N, Navarro MA, Surra JC, Arnal C, Fernandez-Juan M, et al (2007) Cloning, characterization, expression and comparative analysis of pig Golgi membrane sphingomyelin synthase 1. Gene 388: 117–124. 17156943 10.1016/j.gene.2006.10.013

[44] Liu J, Zhang H, Li Z, Hailemariam TK, Chakraborty M, et al (2009) Sphingomyelin synthase 2 is one of the determinants for plasma and liver sphingomyelin levels in mice. Arterioscler Thromb Vasc Biol 29: 850–856. 19286635 10.1161/ATVBAHA.109.185223 PMC2763553

[45] Li Z, Fan Y, Liu J, Li Y, Huan C, et al (2012) Impact of sphingomyelin synthase 1 deficiency on sphingolipid metabolism and atherosclerosis in mice. Arterioscler Thromb Vasc Biol 32: 1577–1584. 22580896 10.1161/ATVBAHA.112.251538 PMC3444302

[46] Geelen MJ, van Hoorn D, Beynen AC (1999) Consumption of casein instead of soybean protein produces a transient rise in the concentration of sphingomyelin in VLDL in rats. J Nutr 129: 2119–2122. 10573537 10.1093/jn/129.12.2119

[47] Bladergroen BA, Beynen AC, Geelen MJ (1999) Dietary pectin lowers sphingomyelin concentration in VLDL and raises hepatic sphingomyelinase activity in rats. J Nutr 129: 628–633. 10082766 10.1093/jn/129.3.628

[48] Geelen MJ, Tijburg LB, Bouma CJ, Beynen AC (1995) Cholesterol consumption alters hepatic sphingomyelin metabolism in rats. J Nutr 125: 2294–2300. 7666245 10.1093/jn/125.9.2294

[49] Geelen MJ, Beynen AC (2000) Consumption of olive oil has opposite effects on plasma total cholesterol and sphingomyelin concentrations in rats. Br J Nutr 83: 541–547. 10953679

[50] Ottestad I, Hassani S, Borge GI, Kohler A, Vogt G, et al (2012) Fish Oil Supplementation Alters the Plasma Lipidomic Profile and Increases Long-Chain PUFAs of Phospholipids and Triglycerides in Healthy Subjects. PLoS One 7: e42550. 22952598 10.1371/journal.pone.0042550 PMC3429454

[51] Bagdade JD, Buchanan WE, Levy RA, Subbaiah PV, Ritter MC (1990) Effects of omega-3 fish oils on plasma lipids, lipoprotein composition, and postheparin lipoprotein lipase in women with IDDM. Diabetes 39: 426–431. 2318345 10.2337/diab.39.4.426

[52] Nilsson A, Duan RD (2006) Absorption and lipoprotein transport of sphingomyelin. J Lipid Res 47: 154–171. 16251722 10.1194/jlr.M500357-JLR200

[53] Subbaiah PV, Liu M (1993) Role of sphingomyelin in the regulation of cholesterol esterification in the plasma lipoproteins. Inhibition of lecithin-cholesterol acyltransferase reaction. J Biol Chem 268: 20156–20163. 8376375

[54] Fournier N, Paul JL, Atger V, Cogny A, Soni T, et al (1997) HDL phospholipid content and composition as a major factor determining cholesterol efflux capacity from Fu5AH cells to human serum. Arterioscler Thromb Vasc Biol 17: 2685–2691. 9409243 10.1161/01.atv.17.11.2685

[55] Nuno-Ayala M, Guillen N, Navarro MA, Lou-Bonafonte JM, Arnal C, et al (2010) Cysteinemia, rather than homocysteinemia, is associated with plasma apolipoprotein A-I levels in hyperhomocysteinemia: lipid metabolism in cystathionine beta-synthase deficiency. Atherosclerosis 212: 268–273. 20537649 10.1016/j.atherosclerosis.2010.04.028

[56] Terpstra AH, Woodward CJ, Sanchez-Muniz FJ (1981) Improved techniques for the separation of serum lipoproteins by density gradient ultracentrifugation: visualization by prestaining and rapid separation of serum lipoproteins from small volumes of serum. Anal Biochem 111: 149–157. 6165257 10.1016/0003-2697(81)90243-8

[57] Kelley JL, Kruski AW (1986) Density gradient ultracentrifugation of serum lipoproteins in a swinging Bucket rotor. Methods in Enzymology 128: 170–181. 3724501 10.1016/0076-6879(86)28067-2

[58] Chapman MJ (1986) Comparative analysis of mammalian plasma lipoproteins. Methods in Enzymology 128: 70–143. 3523143 10.1016/0076-6879(86)28063-5

[59] Bradford MM (1976) A rapid and sensitive method for the quantitation of microgram quantities of protein utilizing the principle of protein-dye binding. Anal Biochem 72: 248–254. 942051 10.1016/0003-2697(76)90527-3

[60] Merril CR, Goldman D, Van Keuren ML (1984) Gel protein stains: silver stains. Methods in Enzymology 104: 441–447. 6201698 10.1016/s0076-6879(84)04111-2

[61] Calleja L, Paris MA, Paul A, Vilella E, Joven J, et al (1999) Low-cholesterol and high-fat diets reduce atherosclerotic lesion development in ApoE-knockout mice. Arterioscler Thromb Vasc Biol 19: 2368–2375. 10521366 10.1161/01.atv.19.10.2368

[62] Navarro MA, Carpintero R, Acin S, Arbones-Mainar JM, Calleja L, et al (2005) Immune-regulation of the apolipoprotein A-I/C-III/A-IV gene cluster in experimental inflammation. Cytokine 31: 52–63. 15878672 10.1016/j.cyto.2005.03.002

[63] Hojjati MR, Jiang XC (2006) Rapid, specific, and sensitive measurements of plasma sphingomyelin and phosphatidylcholine. J Lipid Res 47: 673–676. 16371647 10.1194/jlr.D500040-JLR200

[64] He X, Chen F, McGovern MM, Schuchman EH (2002) A fluorescence-based, high-throughput sphingomyelin assay for the analysis of Niemann-Pick disease and other disorders of sphingomyelin metabolism. Anal Biochem 306: 115–123. 12069422 10.1006/abio.2002.5686

[65] Guillen N, Acin S, Navarro MA, Perona JS, Arbones-Mainar JM, et al (2008) Squalene in a sex-dependent manner modulates atherosclerotic lesion which correlates with hepatic fat content in apoE-knockout male mice. Atherosclerosis 196: 558–564. 17854812 10.1016/j.atherosclerosis.2007.08.008

[66] Arbonés-Mainar JM, Navarro MA, Acín S, Guzmán MA, Arnal C, et al (2006) trans-10, cis-12- and cis-9, trans-11-Conjugated Linoleic Acid Isomers Selectively Modify HDL-Apolipoprotein Composition in Apolipoprotein E Knockout Mice. J Nutr 136: 353–359. 16424111 10.1093/jn/136.2.353

[67] Bustin SA (2010) Why the need for qPCR publication guidelines?–The case for MIQE. Methods. 2009/12/23 ed. pp. 217–226. 10.1016/j.ymeth.2009.12.006 20025972

