# Supplementary material for: Postprandial Changes in High Density Lipoproteins in Rats Subjected to Gavage Administration of Virgin Olive Oil
Source: PLoS One. 2013 Jan 29;8(1):e55231. doi: 10.1371/journal.pone.0055231 (PMC3558467; doi:10.1371/journal.pone.0055231)
Supplement: Table S1 — Fatty acid composition of virgin olive oil. (DOCX) [file pone.0055231.s001.docx]

# Table S1**.** Fatty acid composition of virgin olive oil

| Fatty acids | % |
| --- | --- |
| Lauric (12:0) | -- |
| Myristic (14:0) | 0.1 |
| Palmitic (16:0) | 11.0 |
| Palmitoleic (16:1) | 0.8 |
| Stearic (18:0) | 2.6 |
| Oleic (18:1) | 75.3 |
| Linoleic (18:2n-6) | 8.8 |
| Linolenic (18:3n-3) | 0.6 |
| Arachidic (20:0) | 0.4 |
| Gadoleic (20:1) | 0.3 |
| Behenic (22:0) | 0.1 |

Data are expressed in g/100g
