# Supplementary material for: Postprandial Changes in High Density Lipoproteins in Rats Subjected to Gavage Administration of Virgin Olive Oil
Source: PLoS One. 2013 Jan 29;8(1):e55231. doi: 10.1371/journal.pone.0055231 (PMC3558467; doi:10.1371/journal.pone.0055231)
Supplement: Table S2 — Nucleotide sequence of primers used for RT-qPCR according to MIQE guidelines. (DOCX) [file pone.0055231.s002.docx]

Table S2. Nucleotide sequence of primers used for RT-qPCR according to MIQE guidelines

| \| **Gene** \| **Accession #** \|  \| **Sequence** \| **Junction** \| **[primer]** \| **Efficiency** \| **Amplicon length** \| \| --- \| --- \| --- \| --- \| --- \| --- \| --- \| --- \| \| *Abca1* \| NM_178095 \| sense \| TCGGCTGGTATCGATTTCACA \| exon 10/11 \| 100 nM \| 96% \| 130 \| \|  \|  \| antisense \| GGTCCCAGTACCCATCCTTGAT \|  \|  \|  \|  \| \| *Abcg1* \| NM_053502 \| sense \| TCTGACCTTTCCCCTCGAGAT \| exon 11/13 \| 100 nM \| 100% \| 146 \| \|  \|  \| antisense \| AGTACACGATGCTGCAGTAGGC \|  \|  \|  \|  \| \| *Apoa1* \| NM_012738 \| sense \| GGCAGAGACTATGTGTCCCAGTTT \| exon 3/4 \| 100 nM \| 100% \| 91 \| \|  \|  \| antisense \| TTGAACCCAGAGTGTCCCAGTT \|  \|  \|  \|  \| \| *Apoa1bp* \| NM_001106440 \| sense \| CGGCTATTGCCAAGGCTTAT \| exon 1/2 \| 100 nM \| 94% \| 123 \| \|  \|  \| antisense \| AAAGTTTGAGGTGTCGCGCA \|  \|  \|  \|  \| \| *Apoa2* \| NM_013112 \| sense \| GCCTAGAAGGAGCTTTGGTTCG \| exon 1/2 \| 100 nM \| 98% \| 141 \| \|  \|  \| antisense \| TGGCTTGGTTCTGAATCTCTGA \|  \|  \|  \|  \| \| *Apoa4* \| NM_012737 \| sense \| ACCCTCTTCCAGGACAAACTTG \| exon 3 \| 100 nM \| 96% \| 103 \| \|  \|  \| antisense \| CCTTGGTTAGATGTCCACTCAGTTG \|  \|  \|  \|  \| \| *Apoe* \| NM_138828 \| sense \| CTTGTTTCGGAAGGAGCTGACT \| exon 2 \| 100 nM \| 96% \| 89 \| \|  \|  \| antisense \| AGGCATCCTGTCAGCAATGTG \|  \|  \|  \|  \| \| *Cidec* \| NM_001024333 \| sense \| TCACTGTCCAGGCATGTAGCA \| exon 2/3 \| 100 nM \| 99% \| 127 \| \|  \|  \| antisense \| CCTTTGCGAACCTTCCGAT \|  \|  \|  \|  \| \| *Hprt* \| NM_012583 \| sense \| TCCCAGCGTCGTGATTAGTGA \| exon 1/3 \| 100 nM \| 97% \| 152 \| \|  \|  \| antisense \| CCTTCATGACATCTCGAGCAAG \|  \|  \|  \|  \| \| *Lcat* \| NM_017024 \| sense \| GGCTGTGCTACCGAAAGACAGA \| exon 2/3 \| 100 nM \| 97% \| 105 \| \|  \|  \| antisense \| GACAACCCTGGTGTTATCAATCCA \|  \|  \|  \|  \| \| *Pla2g7* \| NM_001009353 \| sense \| GCGTTTGTACTACCCAGCTCAAGA \| exon 4/5 \| 100 nM \| 99% \| 153 \| \|  \|  \| antisense \| TGCAGGAGTTGTCAGAGAACCA \|  \|  \|  \|  \| \| *Pltp* \| NM_001168543 \| sense \| GTTGAATGAGCGTATCTGGCGT \| exon 14/16 \| 100 nM \| 99% \| 101 \| \|  \|  \| antisense \| CAACAGTGACGAAGCCTGCAT \|  \|  \|  \|  \| \| *Pon1* \| NM_032077 \| sense \| GGACTGGTGTTGGCACTTTACA \| exon 1/3 \| 100 nM \| 98% \| 124 \| \|  \|  \| antisense \| CACCCGCTTCGATTCCTTTA \|  \|  \|  \|  \| \| *Rn18s* \| X01117 \| sense \| ACTCAACACGGGAAACCTCA \| exon 5 \| 100 nM \| 99% \| 114 \| \|  \|  \| antisense \| TCTTAGTTGGTGGAGCGATT \|  \|  \|  \|  \| \| *Scarb1* \| NM_031541 \| sense \| TCAAGAATGTCCGCATAGACCC \| exon 1/2 \| 100 nM \| 98% \| 128 \| \|  \|  \| antisense \| TTCTGGCCATTTAGGACCTCG \|  \|  \|  \|  \| \| *Sgms1* \| NM_181386 \| sense \| CGGGTGCAGTGGGCGTTTTCTATT \| Exón 6/7 \| 100 nM \| 101% \| 198 \| \|  \|  \| antisense \| ACAGTTGAAGTGCATGCCGGGT \|  \|  \|  \|  \| \| *Syt1* \| NM_001033680 \| sense \| CAGAATAACCAGCTGTTGGTGG \| exon 6/7 \| 100 nM \| 96% \| 109 \| \|  \|  \| antisense \| TTTTGTCAGGCAGCAGGAAG \|  \|  \|  \|  \| \| *Tbp* \| NM_001004198 \| sense \| TAATCCCAAGCGGTTTGCTG \| exon 4/6 \| 100 nM \| 92% \| 111 \| \|  \|  \| antisense \| TTCTTCACTCTTGGCTCCTGTG \|  \|  \|  \|  \| \| *Ubc* \| NM_017314 \| sense \| ATCTAGAAAGAGCCCTTCTTGTGC \| exon 3 \| 100 nM \| 98% \| 51 \| \|  \|  \| antisense \| ACACCTCCCCATCAAACCC \|  \|  \|  \|  \| |
| --- | --- | --- | --- | --- | --- | --- | --- | --- | --- | --- | --- | --- | --- | --- | --- | --- | --- | --- | --- | --- | --- | --- | --- | --- | --- | --- | --- | --- | --- | --- | --- | --- | --- | --- | --- | --- | --- | --- | --- | --- | --- | --- | --- | --- | --- | --- | --- | --- | --- | --- | --- | --- | --- | --- | --- | --- | --- | --- | --- | --- | --- | --- | --- | --- | --- | --- | --- | --- | --- | --- | --- | --- | --- | --- | --- | --- | --- | --- | --- | --- | --- | --- | --- | --- | --- | --- | --- | --- | --- | --- | --- | --- | --- | --- | --- | --- | --- | --- | --- | --- | --- | --- | --- | --- | --- | --- | --- | --- | --- | --- | --- | --- | --- | --- | --- | --- | --- | --- | --- | --- | --- | --- | --- | --- | --- | --- | --- | --- | --- | --- | --- | --- | --- | --- | --- | --- | --- | --- | --- | --- | --- | --- | --- | --- | --- | --- | --- | --- | --- | --- | --- | --- | --- | --- | --- | --- | --- | --- | --- | --- | --- | --- | --- | --- | --- | --- | --- | --- | --- | --- | --- | --- | --- | --- | --- | --- | --- | --- | --- | --- | --- | --- | --- | --- | --- | --- | --- | --- | --- | --- | --- | --- | --- | --- | --- | --- | --- | --- | --- | --- | --- | --- | --- | --- | --- | --- | --- | --- | --- | --- | --- | --- | --- | --- | --- | --- | --- | --- | --- | --- | --- | --- | --- | --- | --- | --- | --- | --- | --- | --- | --- | --- | --- | --- | --- | --- | --- | --- | --- | --- | --- | --- | --- | --- | --- | --- | --- | --- | --- | --- | --- | --- | --- | --- | --- | --- | --- | --- | --- | --- | --- | --- | --- | --- | --- | --- | --- | --- | --- | --- | --- | --- | --- | --- | --- | --- | --- | --- | --- | --- | --- | --- | --- | --- | --- | --- | --- | --- | --- | --- | --- | --- | --- | --- | --- | --- | --- | --- | --- | --- | --- | --- | --- | --- | --- | --- | --- | --- | --- | --- | --- | --- |
